# The Impact of Online Dating on External Validation and Self-Perception

**DOI:** 10.1192/j.eurpsy.2025.1432

**Published:** 2025-08-26

**Authors:** M. K. Khamassi, Y. Trabelsi, A. Mtiraoui, J. Nakhli

**Affiliations:** 1 Research Laboratory LR12ES04, University of Sousse, Faculty of Medecine of Sousse; 2 Department of Psychiatry, Farhat Hached Hospital, Sousse, Tunisia

## Abstract

**Introduction:**

In the digital era, online dating platforms have emerged as a prominent means for establishing social relationships. While these platforms facilitate connections, they also introduce a distinct form of external validation through likes, matches, and compliments. Despite their popularity, researchs on the psychological effects of this validation on users are limited.

**Objectives:**

This study aimed to reveal the profile of dating platforms users and assess whether these platforms foster a dependence on external validation and affect operator’s self-esteem

**Methods:**

A cross-sectional study was shared via social media and online forums. In addition to socio demographic and work related variables, it comprised an evaluation of dependency on external validation through a series of targeted questions, and explored its relationship with the use of dating platform. The Rosenberg Self-Esteem Scale was used to assess participants’ self-esteem, with a score below 31 indicating low self-esteem.

**Results:**

The study included 55 participants aged between 19 and 40 years, with a mean age of 26.27. The gender distribution was nearly equal, with 49.1% male (n=27) and 50.9% female(n=28). Most participants (54.54%) were single, and 40% reported using dating apps primarily for casual dating and entertainment.

Among applications users, 46.7% engaged with the platforms several times a week, while 20% used them daily. Furthermore, 53.7% of users plan to continue using them.

Regarding external validation, 64.4% reported that receiving validation motivated them to use the applications more frequently, while 56.2% indicated they would adjust their behavior based on feedbacks. Additionally, 47.1% felt influenced by compliments, and 58.9% reported that criticism and rejection affected them negatively.

According to the Rosenberg Self-Esteem Scale, 33.3% of users exhibited low self-esteem and 28.5% of users reported that dating applications had a negative impact on their self-esteem. Notably, no significant correlation was found between dating applications’ usage and self-esteem (p = 0.53 > 0.05).

**Conclusions:**

This study highlights a reliance on external validation among online dating users, suggesting that these platforms may encourage behaviors driven by the need for approval. This underscores the importance of further research into the psychological effects of such dependence.

**Disclosure of Interest:**

None Declared

